# Prevalence and patterns of maxillofacial trauma: a retrospective descriptive study

**DOI:** 10.1007/s00068-019-01174-6

**Published:** 2019-06-21

**Authors:** Ammar Al-Hassani, Khalid Ahmad, Ayman El-Menyar, Ahmad Abutaka, Ahammed Mekkodathil, Ruben Peralta, Moustafa Al Khalil, Hassan Al-Thani

**Affiliations:** 1 Department of Surgery, Trauma Surgery Section, Hamad Medical Corporation, Doha, Qatar; 2 Clinical Research, Trauma and Vascular Surgery Section, Hamad Medical Corporation, PO Box 3050, Doha, Qatar; 3 Clinical Medicine, Weill Cornell Medical College, Doha, Qatar; 4 Department of Surgery, Maxillofacial Surgery, Hamad Medical Corporation, Doha, Qatar

**Keywords:** Maxillofacial injury, Road traffic accident, Trauma, Mandibular, Qatar

## Abstract

**Introduction:**

We aimed to describe the prevalence and pattern of maxillofacial trauma in Qatar.

**Methods:**

This is a retrospective study of trauma registry data at Hamad General Hospital during the period from January 2011 to December 2014. The study included all traumatic maxillofacial patients who underwent CT scan and were admitted during the study period.

**Results:**

A total of 1187 patients with maxillofacial injuries were included in the study and 18.5% of all trauma admissions were related to maxillofacial injuries. Young age and males were predominantly affected. Mechanisms of injury were mainly traffic-related and fall. Orbital injuries were the commonest followed by maxillary injuries. The median and range face abbreviated injury score (AIS) was 2 [1–3] with 66% had a score of 2. Maxillofacial fractures were frequently associated with traumatic brain injuries. One out of five patients was managed with surgery and had median length of stays in ICU and hospital 5 and 7 days, respectively. Overall, in-hospital mortality was 8.3%. Mortality in isolated maxillofacial was low (0.3%) in comparison to 15% in polytrauma patients (*p* = 0.001). Multivariable regression analysis showed that Injury Severity Score, face AIS and Glasgow Coma Scale were predictors of mortality with age-adjusted odd ratio of 1.15, 2.48 and 0.82; respectively.

**Conclusions:**

Maxillofacial trauma requiring admission is not uncommon in our trauma center and mostly it is mild to moderate in severity. Associated injuries are present in most of the maxillofacial injured patients and further diagnostic investigations should be part of the assessment in maxillofacial injuries.

## Introduction

Maxillofacial (MF) injuries constitute one of the major health problems worldwide. These injuries remain as a serious clinical problem because of the sensitivity of this anatomical region [1]. Although these injuries are common worldwide, their incidence and pattern are of major concern since it is linked with several factors including social, cultural, and environmental factors and, therefore, varies with population [1–4]. Road crashes remain as the main cause of MF injuries, followed by assaults, sports, occupational-related injuries, and falls [2–6]. Motor vehicle crashes (MVCs) were the predominant cause in the GCC (Gulf Cooperation Council) countries [7]. Most of MF fractures occur in males between the ages of 21 and 30 years, with male-to-female ratio ranges from 2:1 to 11:1 [2, 5–7]. It is often associated with substantial morbidity, deformity, loss of function, and high treatment cost [3].

In MVCs, patients are more likely to get injured while speeding and not wearing a seat belt. Soft tissue injuries were the most common associated injuries, and in the past conventional plain, radiography was the usual investigation [8]. Mandible was seen as the most predominantly fractured bone in multiple studies. Careful inspection, palpation, and examination of function assure accurate diagnosis of the injuries. Management of MF trauma has developed in an evolutionary manner. Evaluation of injuries of soft tissue and bone must be precise through instrumental diagnostic examinations. Coordinated, periodic, and sequential collection of data concerning demographic patterns of MF injuries may assist health care officials assess address the causes and evaluate effectiveness of previously implemented preventive protocols. Consequently, an understanding of the etiology, severity, temporal distribution, and prevalence of MF trauma may dictate priorities to be implemented on the basis of the findings [2].

Pattern of MF injuries in Qatar remains understudied. The present study aims to describe the prevalence and pattern traumatic MF injury in the only level 1 trauma center in Qatar. The study will address the existing gaps in knowledge in Qatar and ultimately contribute to the evaluation of existing preventive strategies and development of new measures in injury prevention, whenever applicable. Furthermore, insight into the epidemiology of facial fractures and concomitant injuries is an integral component in evaluating the quality of patient care, developing optimal treatment regimens, and making decisions.

## Methods

Ethical approval was granted from the medical research center and institutional review board (IRB# 15184/15) and then data were retrieved from the trauma registry database. Maxillofacial trauma refers to any injury to the face or jaw caused by physical force, foreign objects. Data were acquired retrospectively for all trauma patients identified from the trauma registry database who were admitted to the section of trauma surgery at Hamad general hospital (HGH) between January 2011 and December 2014. HGH is the national Level 1 trauma center facility in Qatar which admits and treats all traumatic injury patients in the country.

Our study included trauma patients with MF injuries who were diagnosed by computed tomography (CT scan) scan. Patients with non-traumatic MF injuries and those who had not undergone CT scan were excluded.

On arrival, all patients underwent thorough clinical assessment and resuscitation according to Advanced Trauma Life Support (ATLS) guidelines. Treatment by surgery or non-surgically is based on the MF surgeon evaluation and decision. Collected data included age, gender, mechanism of injury, location of MF injuries, associated injuries (brain, skull, cervical spine, soft tissue, chest, abdomen, pelvis, and extremities), injury characteristics, surgical interventions, intubation and outcomes(ICU days, ventilator days, hospital length of stay and mortality). Injury characteristics including Glasgow Coma Score (GCS) at ED, Abbreviated Injury Scale (AIS) and Injury Severity Score (ISS) were collected. AIS scoring is an anatomical-based coding system created by the Association for the Advancement of Automotive Medicine to classify and describe the severity of injuries. The Injury Severity Score (ISS) is an established medical score to assess trauma severity. It correlates with mortality, morbidity and hospitalization time after trauma.

Patients were grouped based on their mechanism of injury such as MVC, pedestrian and falls. Comparative analysis was performed. Patients were also stratified based on ISS < 15 and ISS ≥ 15 and compared for the characteristics.

### Statistical analysis

Data were expressed as proportions, medians, or mean ± standard deviation (SD), as appropriate. Differences in categorical variables between respective comparison groups were analyzed using Chi square test or Fisher exact (observed cell values less than 5) test for categorical variables. The continuous variables were analyzed using student’s *t* test. Two-tailed *p* values < 0.05 were considered to be significant. Multivariate regression analysis was performed to look for the predictors of mortality using the most relevant variables (age, systolic blood pressure (SBP), GCS, ISS, face AIS and intubation) and data were expressed as odd ratio (OR) and 95% confidence interval (CI). Data analysis was carried out using the Statistical Package for Social Sciences version 18 (SPSS Inc. Chicago, Illinois, USA).

## Results

**Fig. 1:**
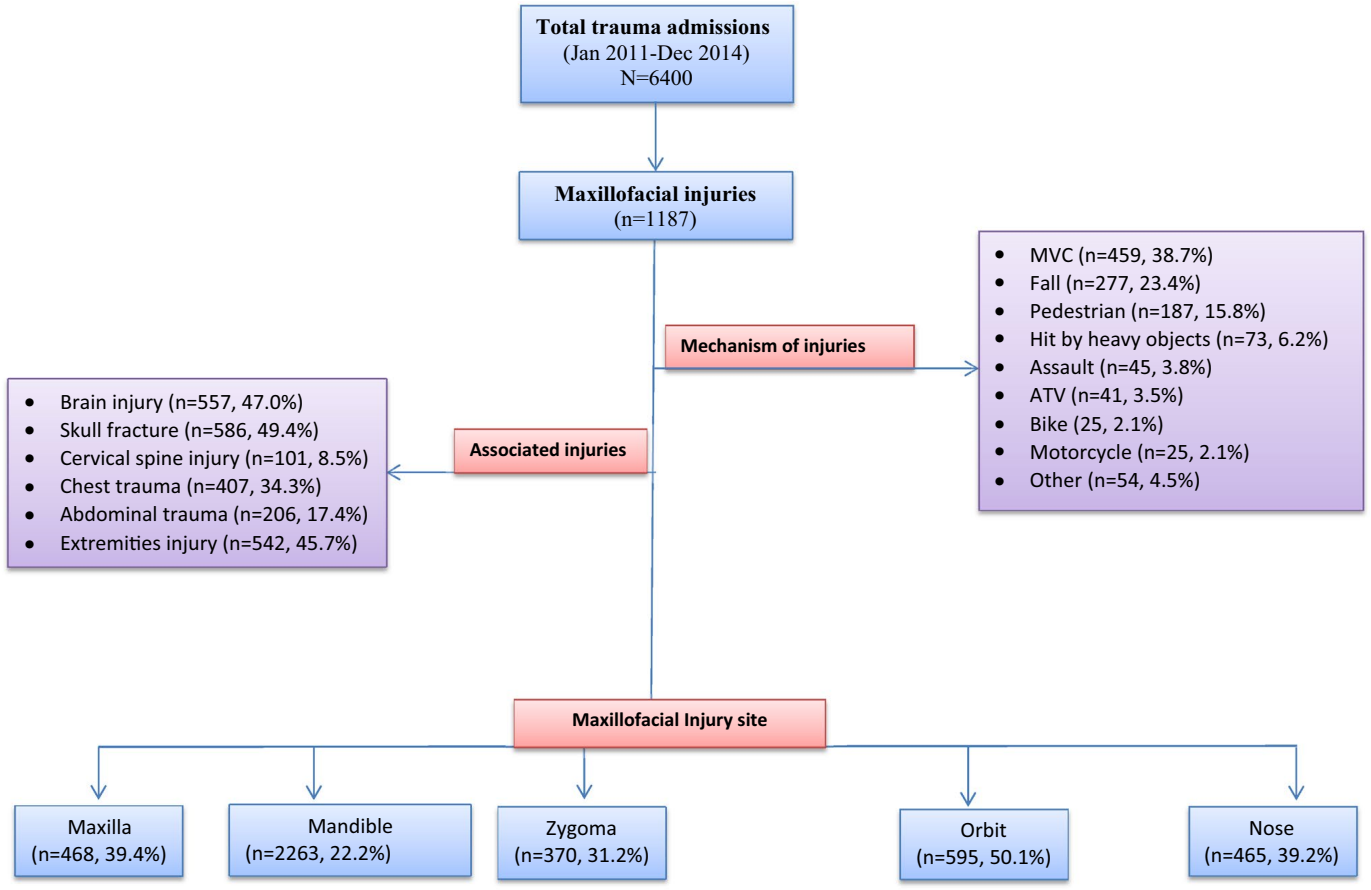
Study design, associated injury and mechanism of injury

A total of 1187 patients with MF injuries were included in the study (Table 1). The mean age of the patients was 31.4 ± 14.0 with a male predominance (93%). The most common age group involved in MF trauma was the third decade of life (33%) followed by the fourth decade (22%). MF trauma in children and elderly was very rare. Figure 1 describes the study design and injuries. The mechanism of injury was MVC (39%) in the majority of patients followed by fall (23%) whereas pedestrians hit by vehicles were 16%.

**Table 1:** Demographic, injury characteristics, interventions and outcomes of patients with maxillofacial injuries (*N* = 1187)

Age (mean ± SD)	31.4 ± 14.0
Age groups	
0–10	55 (4.7%)
11–20	180 (15.5%)
21–30	388 (33.4%)
31–40	253 (21.8%)
41–50	183 (15.8%)
51–60	64 (5.5%)
> 60	38 (3.3%)
Males	1106 (93.2%)
Face AIS score [median (range)]	2 (1–3)
AIS = 1	387 (33.0%)
AIS = 2	769 (65.7%)
AIS = 3	15 (1.3%)
Glasgow Coma Score (mean ± SD)	11.6 ± 3.4
Injury Severity Score (mean ± SD)	17.6 ± 10.2
Surgical intervention	223 (18.8%)
Intubation	449 (37.8%)
ICU LOS [median (range)]	5 (1–155)
Total LOS [median (range)]	7 (1–304)
Mortality	98 (8.3%)

*SD* standard deviation, *AIS* Abbreviated Injury Score, *ICU* Intensive Care Unit, *LOS* length of stay

Orbital fractures (50%) account for the majority of the cases followed by maxillary fractures (39%) and nasal fractures (39%). Zygomatic fractures were present in 31% and mandibular fractures (ramus, body and condylar fractures) accounted for 22.5%.

Associated injuries include; skull base fracture (49%) and traumatic brain injuries (47%). Due to the polytrauma nature of the injuries, chest, abdomen and extremities were involved in 34%, 17% and 46%, respectively. Cervical spine injuries were present only in 9%.

Mean AIS score for the face was 2 in the majority (66%); 1 in 33% and was 3 in 15 (1.3%) patients.

Mean ISS was 17.6 ± 10.2. Mean GCS on arrival was 11.6 ± 3.4. Thirty-eight percent underwent intubation and 19% had surgical intervention. The median ICU length of stay (LOS) and hospital LOS were 5 and 7 days, respectively. In-hospital mortality was reported in 98 (8.3%) cases in which 83% were having associated brain injury and 98% had ISS ≥ 15.

Table 2 summarizes the demographic information, injury characteristics, interventions and outcomes in patients with MF trauma by mechanism of injuries such as MVCs, pedestrian hit by vehicle and falls.

**Table 2:** Injury characteristics, interventions and outcomes of patients with maxillofacial injuries by mechanism of trauma

	MVC (*n* = 459)	Pedestrian (*n* = 187)	Fall (*n* = 277)	*P*
Age (mean ± SD)	28.9 ± 12.8	36.3 ± 15.7	31.8 ± 14.9	0.001
Males (%)	409 (89.1)	179 (95.7)	265 (95.7)	0.001
Injury sites; *n* (%)				
Maxillary	192 (41.8)	77 (41.2)	91 (32.9)	0.04
Mandibular	103 (22.4)	40 (21.4)	59 (21.3)	0.6
Zygomatic	127 (27.7)	64 (34.2)	88 (31.8)	0.2
Orbit	215 (46.8)	92 (49.2)	145 (52.3)	0.4
Nose	193 (42.0)	69 (36.9)	95 (34.3)	0.1
Associated injuries, *n* (%)				
Brain injury	210 (45.7)	105 (56.1)	132 (47.7)	0.04
Skull fracture	214 (46.6)	97 (51.9)	151 (54.5)	0.2
Cervical spine	54 (11.8)	15 (8.0)	19 (6.9)	0.07
Chest	199 (43.4)	85 (45.5)	70 (25.3)	0.001
Abdomen	101 (22.0)	37 (19.8)	41 (14.8)	0.06
Extremities	233 (50.8)	91 (48.7)	139 (50.2)	0.9
Face AIS [median (range)]	2 (1–3)	2 (1–3)	2 (1–3)	0.8
Glasgow Coma Score (mean ± SD)	10.9 ± 4.0	10.7 ± 4.3	12.3 ± 2.7	0.001
Injury Severity Score (mean ± SD)	18.9 ± 10.4	19.0 ± 10.1	17.1 ± 9.0	0.04
Surgical intervention (*n*, %)	90 (19.6)	24 (12.8)	49 (17.7)	0.1
Intubation (*n*, %)	209 (45.5)	82 (43.9)	84 (30.3)	0.01
ICU LOS [median (range)]	6.0 (1–155)	4 (1–150)	5 (1–36)	0.1
Total LOS [median (range)]	9.0 (1–304)	7 (1–211)	7 (1–223)	0.1
Mortality; *n* (%)	48 (10.5)	25 (13.4)	13 (4.7)	0.03

*SD* standard deviation, *AIS* Abbreviated Injury Score, *ICU* Intensive Care Unit, *LOS* length of stay

There were significant differences in age of the patients by mechanism of injury; patients in the MVC group were younger (29 years) compared to other MOI groups (*p* = 0.001). In all groups, males were predominant (*p* = 0.001).

Maxillary injuries were more common in the MVC group (42%) when compared to other groups (*p* < 0.05). However, injuries to other sites such as mandibular, zygomatic, orbit and nose were comparable.

Brain injuries were more frequent in pedestrian group (56%) than the other two groups, though its incidence also were relatively high in the other group around 46–48% (*p* = 0.04). On the other hand, skull fracture was comparable across the groups. In addition, chest injuries were more common in the pedestrian group (*p* = 0.001), whereas injuries to the cervical spine, abdomen and extremities were comparable.

The face AIS across the three groups were not found to be statistically significant; however, ISS score was significantly higher in the pedestrian group (*p* = 0.04). The mean GCS was low in the pedestrian group with mean value 10.7 ± 4.3 (*p* = 0.001).

Intubation was performed more frequently in the MVC group (46%) (*p* = 0.01). There was no statistically significant difference seen in terms of surgical intervention, ICU LOS, and total LOS. Mortality was more common in the pedestrian group (13%) followed by MVC (11%) and fall group (5%) (*p* = 0.03).

Table 3 summarizes the characteristics of the patients based on ISS < 15 and ISS ≥ 15. Nearly 51% of the patients were included in ISS ≥ 15 groups. There were no differences in terms of age or gender distribution across the injury severity groups. MVCs and pedestrian injuries were more likely to result in severe injuries. Maxillary, zygomatic and orbital injuries were more common in ISS ≥ 15 while nasal fractures were more common in ISS < 15 group. All associated injuries such as skull fracture, brain injuries, chest injuries, cervical spine injuries, abdominal trauma and injuries to extremities were significantly higher in severely injured group (in ISS ≥ 15). In addition, ICU LOS, total LOS, intubation and mortality were also high in the ISS ≥ 15 group.

**Table 3:** Patient characteristics, interventions and outcomes of maxillofacial injuries by Injury Severity Score (ISS)

	ISS < 15 (*n* = 582, 49.3%)	ISS ≥ 15 (*n* = 599, 50.7%)	*P*
Age (mean ± SD)	31.4 ± 14.3	31.3 ± 13.7	0.9
Males (*n*, %)	534 (91.8)	566 (94.5)	0.06
Mechanism of injury; *n* (%)			
MVC	205 (35.2)	251 (41.9)	0.02
Pedestrian	73 (12.5)	113 (18.9)	0.003
Fall	138 (23.7)	139 (23.2)	0.8
Injury sites; *n* (%)			
Maxillary	176 (30.2)	290 (48.4)	0.001
Mandibular	128 (22.0)	131 (21.9)	0.8
Zygomatic	135 (23.2)	235 (39.2)	0.001
Orbit	239 (41.1)	356 (59.4)	0.001
Nose	256 (44.0)	207 (34.6)	0.001
Associated injuries; *n* (%)			
Brain injury	147 (25.3)	408 (68.1)	0.001
Skull fracture	181 (31.1)	402 (67.1)	0.001
Cervical spine	26 (4.5)	74 (12.4)	0.001
Chest	75 (12.9)	330 (55.1)	0.001
Abdomen	39 (6.7)	165 (27.5)	0.001
Extremities	183 (31.4)	358 (59.8)	0.001
Glasgow Coma Score (mean ± SD)	13.9 ± 1.0	9.4 ± 5.5	0.001
Surgical intervention; *n* (%)	124 (21.3)	99 (16.5)	0.03
Intubation; *n* (%)	67 (11.5)	376 (62.8)	0.001
ICU length of stay [median (range)]	2 (1–51)	6 (1–155)	0.001
Total length of stay [median (range)]	4 (1–122)	14 (1–304)	0.001
Mortality; *n* (%)	2 (0.3)	90 (15.0)	0.001

We excluded 6 patients with unknown ISS

*SD* standard deviation, *AIS* Abbreviated Injury Score, *ICU* Intensive Care Unit, *LOS* length of stay

### Multivariable regression analysis

To look at the predictors of mortality in patients with MF injury, multivariable regression analysis showed that face AIS, GCS and ISS, were predictors of mortality with age-adjusted odd ratio of 1.15 (95% CI 1.103–1.192), 2.48 (95% CI 1.166–2.276) and 0.82 (95% CI 0.737–0.907); respectively (Table 4).

**Table 4:** Predictors of mortality in patients with maxillofacial injury

Variable	Odd ratio	95% Confidence interval		*P*
Age	1.059	1.035	1.084	0.001
Injury Severity Score	1.147	1.103	1.192	0.001
Face AIS	2.480	1.166	5.276	0.018
SBP at ED	0.994	0.982	1.005	0.260
GCS at ED	0.818	0.737	0.907	0.001
Intubation	1.948	0.413	9.185	0.399

*AIS* Abbreviated Injury Score, *ED* Emergency Department, *GCS* Glasgow Coma Score

## Discussion

The present study revealed that 1187 patients with MF trauma were admitted to the national level 1 trauma center in a 4-year period. The estimated number of all trauma admissions in this center was 1600 per year. Therefore, the present study estimated that 18.5% of all trauma admissions in Qatar were related to MF trauma. Young age (mainly third decade of life) and male gender were predominantly affected. The main mechanisms of injuries were MVCs, falls and pedestrians hit. Orbital injuries were more frequent followed by maxillary injuries. MF fractures are frequently associated with traumatic brain injuries. One out of five patients was managed with surgery (either closed or open reduction) and 4/5 had non-surgical interventions, based on CT findings, examination and MF surgeon’s discretion. MF fractures also had median length of stays in ICU and hospital 5 and 7 days, respectively. Overall, in-hospital mortality was 8.3% that could be related mainly to associated brain injury and higher ISS. Mortality in isolated MF is low (0.3%) in comparison to 15% in polytrauma patients (*p* = 0.001). Our study showed also that Face AIS, GCS and ISS were independent predictors of mortality in MF injury after adjustment for age, SBP and intubation.

Evidence suggests that etiology, incidence and patterns of MF and associated injuries vary with geographic location and socioeconomic status of a population [9]. Therefore, epidemiological data are central and should be taken into account while developing strategies to improve healthcare in a given population [10]. The present study was based on trauma registry data obtained from a national level trauma center which sees and treats all moderate to severe trauma patients across the country.

The majority of the published studies showed MF injuries are common in the age range of 21–30 years which is also shown in our study population [1, 2, 11]. Most of the available literature on MF fractures also revealed that MVC was the most common cause [1, 6, 11, 12] which is also evident in our study. While some of the studies [2, 13] showed mandible fractures as the most common type of MF fractures, our study showed orbital fractures were more common that followed by maxillary fractures This finding was comparable to the previous studies which also showed the same pattern of injuries [12, 14].

This study found that nearly half of the patients had traumatic brain injuries (TBI) and also having skull fractures indicating the severity of trauma which may be reflected on the outcome of the patients with MF fractures. The overall mortality was reported in 98 patients (8%). Previous studies showed TBI as the poor prognostic factor in MF injuries [12, 15].

In the current study, TBI was significantly present in the MF injuries with ISS ≥ 15 which reflect the severity of mechanism of injury. In addition, this group of patients had low GCS as compared to ISS < 15 and was found to be statistically significant. Therefore, it is very important to maintain a high level of suspicion for intracranial lesions in all patients with MF trauma, even those with no obvious signs and symptoms of brain injury. In addition, cervical spine injuries in MF trauma (9%) signify the importance of suspicion for cervical spine injury in isolated MF injuries.

### Limitations

 This is a retrospective study that could have the inherent potential of missing information and selection bias. The MF injury classification and patient management were not described as it was not available in the trauma database and was not the main scope of this paper. Missed injury on follow-up was also not reported in the trauma registry.

## Conclusions

MF trauma requiring admission is not uncommon in our trauma center and mostly it is of mild to moderated severity. Mortality is related mainly to the severity of trauma and associated injuries (AIS and ISS) and GCS. Associated injuries are present in most of the MF-injured patients and further diagnostic investigations should be part of the assessment in MF injuries. This study provides an insight into the epidemiology of maxillofacial fractures and concomitant injury which is an integral component in evaluating the quality of patient care, developing optimal treatment regimens, and making decision.
